# A reverse micelle strategy for fabricating magnetic lipase-immobilized nanoparticles with robust enzymatic activity

**DOI:** 10.1038/s41598-017-10453-4

**Published:** 2017-08-29

**Authors:** Shixiong Yi, Fangyin Dai, Cunyi Zhao, Yang Si

**Affiliations:** 1 State Key Laboratory of Silkworm Genome Biology & College of Biotechnology, Southwest University, Chongqing, 400715 P. R. China; 2 Fiber and Polymer Science, University of California, Davis, CA 95616 USA

## Abstract

Enzyme-immobilized nanoparticles that are both catalysis effective and recyclable would have wide applications ranging from bioengineering and food industry to environmental fields; however, creating such materials has proven extremely challenging. Herein, we present a scalable methodology to create Candida rugosa lipase-immobilized magnetic nanoparticles (L-MNPs) by the combination of nonionic reverse micelle method and Fe_3_O_4_ nanoparticles. Our approach causes the naturally abundant and sustainable Candida rugose lipase to ordered-assemble into nanoparticles with high catalytic activity and durability. The resultant L-MNPs exhibit the integrated properties of high porosity, large surface area, fractal dimension, robust enzymatic activity, good durability, and high magnetic saturation (59 emu g^−1^), which can effectively catalyze pentyl valerate esterification and be easily separated by an external magnet in 60 second. The fabrication of such fascinating L-MNPs may provide new insights for developing functional enzyme-immobilized materials towards various applications.

## Introduction

Enzymes are biocatalysts capable of accelerating a range of chemical transformations relevant to materials production, pharmaceutical development, and renewable energy, which are promising green and sustainable alternative to conventional synthetic strategies^1–4^. In order to adapt to organic reaction media and improve the recovery and reuse efficiency, usually hydrophilic enzymes need to be immobilized on or in specific carriers via physical adsorption or chemical binding^5–7^. Among the numerous supports investigated and applied for enzyme immobilization, nanomaterials, such as nanoparticles, nanotubes, graphene, and nanowires, combine the robust mechanical strength, high porosity, large surface area, lower mass transfer resistance, and high enzyme loading efficiency, which hold great promise as an exceptional nanoscale carrier for realizing enzyme immobilization^8–12^. However, conventional covalent immobilization of enzymes to nanocarrier was performed in aqueous phase, which was usually associated with a significant activity decrease because the active site might be blocked from substrate accessibility and multiple point-binding^13, 14^. The enzyme could also be denatured due to the random cross-linking between proteins and supports^14, 15^.

Reverse micelles, as the forefront of microemulsion reaction medium, has attracted increasing interesting from the viewpoints of avoidance for the random cross-linking between proteins and supports^16, 17^. The reverse micelles are nano-sized spherical aggregates formed by certain surfactants in non-polar medium spontaneously, which solubilized small amounts of water in their interior so providing a stable aqueous microenvironment, the so-called “water-pool”, in non-aqueous medium^18–20^. The enzymes could be interfacial-activated for the oil-water interface and tended to localize at the spherical interface with the active sites orientated towards hydrophobic phase owing to a large associated hydrophobic region^21^. These organized enzymes would then form a self-immobilized particle in the presence of cross-linking agents. Several nanocarriers, including carbon nanotubes, copolymer particles, zeolite, and nanoclay, have recently been applied to enzyme immobilization using reverse micelles synthesis^20–23^. However, previous efforts mainly used these materials as non-functional nanosupports and focused excessively on the loading amount, ignoring the time-consuming and cost enzyme recovery procedures by separation or filtration, which presents major challenges in enzyme immobilization that must be addressed before their extensive practical applications.

Herein, we demonstrate a scalable strategy for creating magnetic lipase-immobilized magnetic nanoparticles (L-MNPs) using a novel nonionic reverse micelle method and Fe_3_O_4_ nanoparticles. The premise of our design is that the *Candida rugosa* lipase are ordered incorporated into Fe_3_O_4_ particles with high catalytic efficiency and durability. The L-MNPs exhibited the integrated properties of high porosity, large surface area, robust enzymatic activity, good durability, and easy to magnetic separation, all originating from the synergistic effect of well-organized *Candida rugose* lipase and magnetic Fe_3_O_4_ nanoparticles.

## Results and Discussion

### Reverse Micelle Design: Optimizing Water Polarity

**Figure 1:**
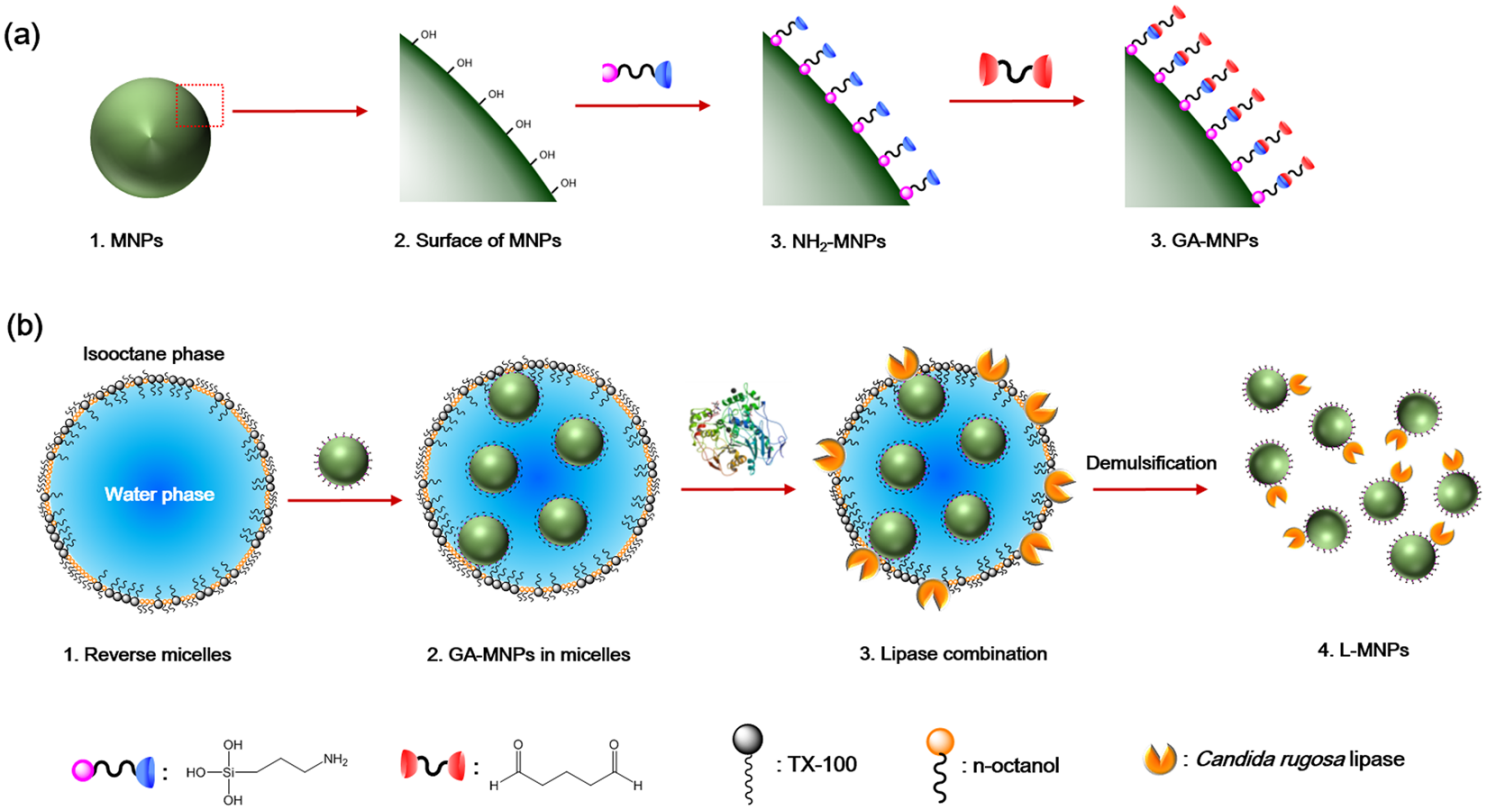
(**a**) Schematic showing the synthetic steps of the GA-MNPs. (**b**) Synthesis of L-MNPs through the nonionic reverse micelle method.

We designed the L-MNPs based on three criteria: (1) the lipase must assemble and bonding to MNPs with a stable covalent linking, (2) the lipase must be exhibit an ordered conformation with high enzymatic bioactivity, and (3) the L-MNPs should be easily collected and separated after reaction. The first two requirements were satisfied by a versatile and readily accessible nonionic reverse micelle method, which allowed quick, easy and reproducible preparation and high structural uniformity of the products. To satisfy the third criterion—we used Fe_3_O_4_ nanoparticles as magnetic nanocarriers for the ordered loading of lipase. The overall synthesis pathway was schematic showing in Fig. 1. The synthesis process began with the reverse micelles medium by optimizing the water polarity. The magnetic Fe_3_O_4_ nanoparticles (MNPs) were first modified with free amino groups on the surface (NH_2_-MNPs), then bonded with glutaric dialdehyde to form active aldehyde modified MNPs (GA-MNPs). Subsequently, the self-immobilization of lipase on GA-MNPs was carried out in an optimized reverse micelles medium, within vigorously stirring by an ultrasonic mixer. Finally, the lipase modified MNPs (L-MNPs) were facilely separated and collected by an external magnetic field.

**Figure 2:**
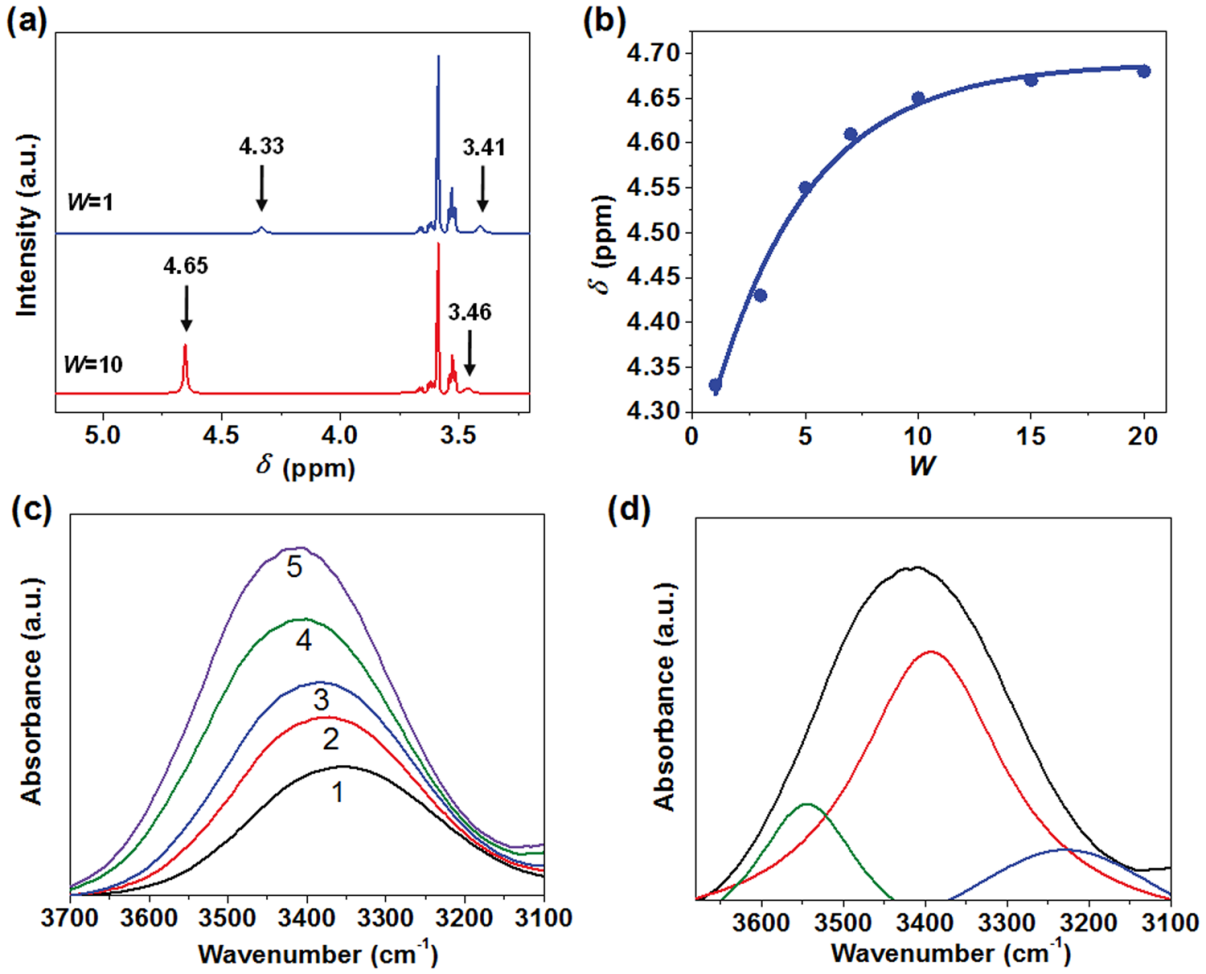
(**a**) ^1^H NMR spectra of TX-100 reverse micelles at *W* = 1 and *W* = 10. (**b**) Chemical shift of –OH protons with different *W* values. (**c**) FT-IR absorption intensify of –OH stretching vibration with different *W* values, from samples 1 to 5: *W* = 0.5, 1.0, 2.0, 5.0, and 20. (**d**) Curve-fitted for FT-IR peak in water hydroxyl in reverse micelles at *W* = 20.

It was well known that the ionic systems such as sodium dioctylsulfosuccinate (AOT) reverse micelles would take remarkable influence on enzyme conformation. Usually enzymes showed lower activity compared to that in an aqueous system, which was attributed to the abnormally high polarity of interface regions of the water pool in an ionic surfactant reverse micelle^24–28^. Therefore, a nonionic reverse micelle system was introduced in this work, and the water polarity was optimized by adjusting the amount of solubilized. The polarity of water in nonionic TX-100 reverse micelles was measured and compared by using^1^H NMR spectra and FT-IR spectra techniques with different *W* values, respectively. Figure 2a and b showed the variation in the NMR chemical shifts of the –OH and –CH_2_CH_2_O– protons with different *W* values in the TX-100 reverse micelles, respectively. It was observed that the chemical shifts of the –OH moves from about 4.33 to 4.65 ppm with increasing *W* values, while the peak of the proton signal of –CH_2_CH_2_O– shifts from 3.41 to 3.46 ppm. The chemical shifts variation of the –OH is due to the changes in the environment of the water molecules^29^. Generally, the hydrogen bonding decreases the electron density around the proton and thus moves the proton absorption to a lower field^25^. The water molecules are initially tightly bound to the ethylene oxide residues, with a low degree of hydrogen bonding per molecule when the *W* = 1. As the amount of water added in the reverse micelles increased and *W* = 10, the hydration process of the water and ethylene oxide was finished and the free water are formed in this system, which caused the increasing hydrogen bonding. Thus the downfield shift in the –OH signal was produced from 4.33 to 4.65 ppm and the –OH chemical shifts was close to the bulk water value of about 4.79 ppm with increasing *W* values^26^. In addition, the small change in the –CH_2_CH_2_O– chemical shift can be ascribed to the changed solvent environment. With the *W* values increased from 1 to 10, the ethylene oxide protons are removed from its initial isooctane environment into the interface of the reverse micelles. Significantly, the^1^H-NMR spectra demonstrated that the polarity of the water added in TX-100 reverse micelles increased with increasing *W* values and get closer to the bulk water, which is of great importance to achieve well enzyme conformation with promising enzymatic activity^30–33^.

This result was further confirmed by FT-IR analysis, as shown in Fig. 2c and d. The total peak area of –OH stretching band of water solubilized in TX-100 reverse micelles changed with different *W* values. The variation of –OH stretching frequencies of water as a function of *W* in this system, where the –OH stretching vibration of water molecules shift from 3348 to 3410 cm^−1^. This is similar to the variation of -OH stretching frequency of water with increasing *W* in the water/AOT/*n*-heptane system. The -OH stretching frequency of 3410 cm^−1^ is the same as that of bulk water. Thus, it was concluded that the water solubilized in reverse micelles behaves analogous the bulk water with increasing *W* values. When the amount of water was small in the reverse micelles, the polarity of water was lower than that in bulk water. It was believed that the polarity of water solubilized in TX-100 reveres micelles at higher *W* values was similar to that in bulk water. The activities of lipase could be remained in TX-100 reverse micelles.

### Morphology and Structure of L-MNPs

**Figure 3:**
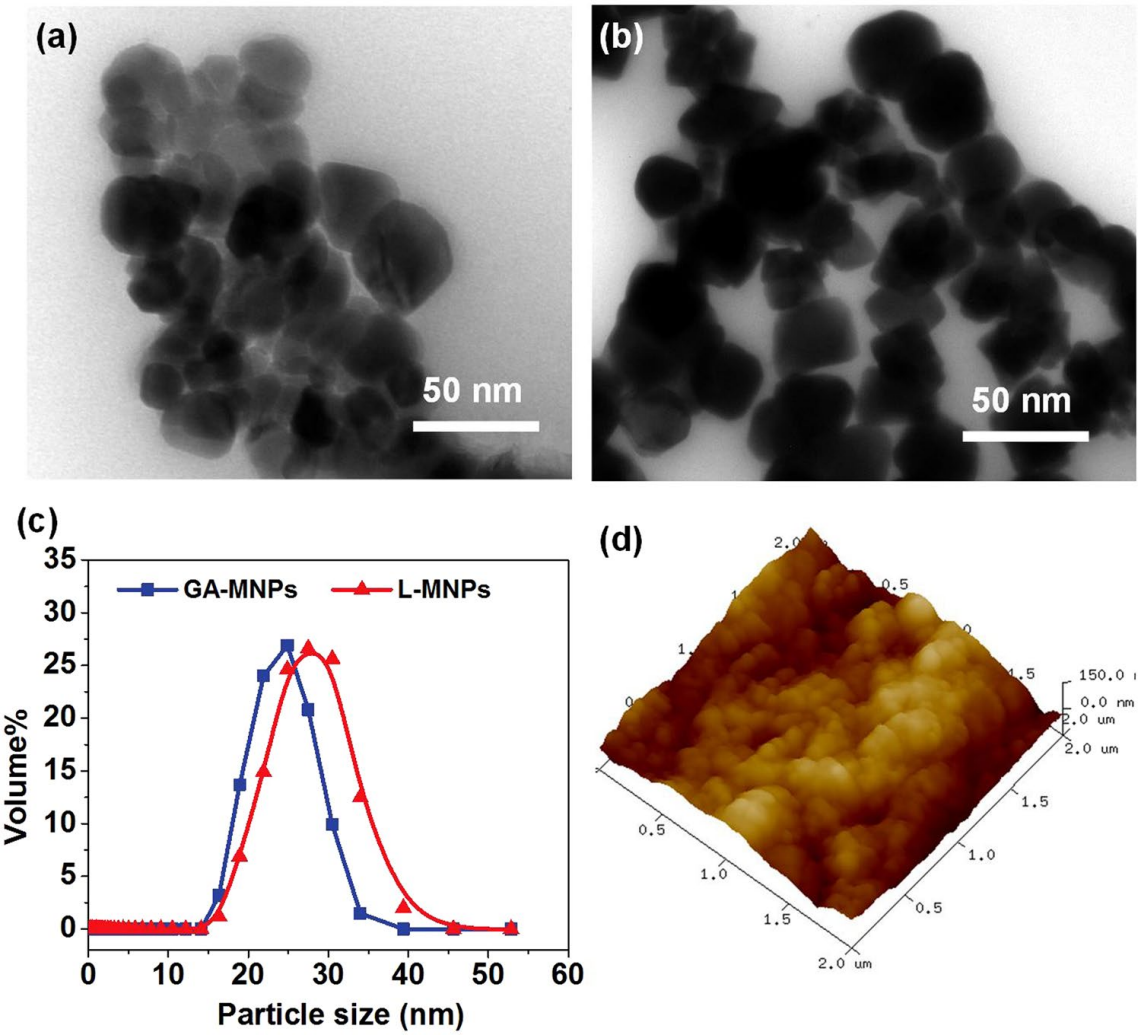
TEM images of (**a**) GA-MNPs and (**b**) L-MNPs. (**c**) Particle size distribution curves of GA-MNPs and L-MNPs. (**d**) AFM topographic image of the L-MNPs in an small area of 2 × 2 μm^2^.

It was shown from Fig. 3a and b that the L-MNPs before and after immobilization was with narrow size distribution by TEM observation. This revealed that the conjugation process did not significantly result in agglomeration and change in size of the nanoparticles, which could be attributed to the fact that the reaction occurred only on the Fe_3_O_4_ nanoparticle surface^34^. Fig. 3c indicated the mean particle diameter of the L-MNPs before and after immobilization was about 25 nm, and most of the L-MNPs had particle sizes of 30–35 nm, which matched well with the TEM results. Moreover, the AFM image also indicated that the preference for particles to self-associate, probably due to magnetic attraction, the absence of a strong interaction with the substrate, and the procedure of sample preparation (Fig. 3d). It is not possible to recognize discrete single enzyme nanoparticle moieties because of the aggregation between particles.

**Figure 4:**
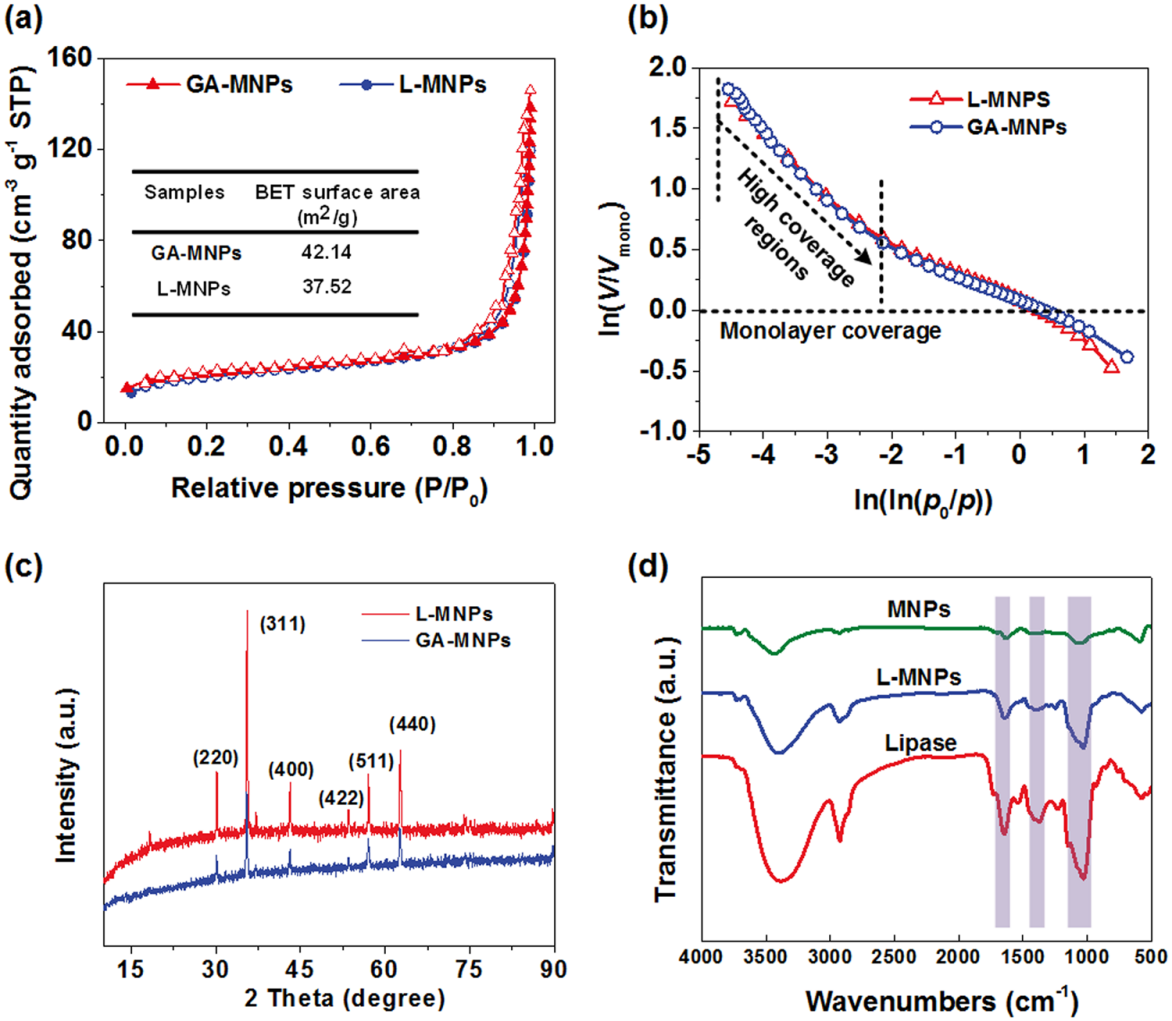
(**a**) N_2_ adsorption−desorption isotherms of L-MNPs before and after immobilization. (**b**) FHH plots of ln(*V*/*V*_mono_) against ln(ln(*p*_0_/*p*)) reconstructed from the relevant N_2_ adsorption isotherms. (**c**) The XRD patterns of GA-MNPs and L-MNPs. (**d**) FT-IR spectra of MNPs, L-MNPs and *Candida rugosa* lipase.

To provide insight into the nanoporous structure, we investigated the effect of lipase immobilization on the rough structure of resultant MNPs through N_2_ adsorption at 77 K. As shown in Fig. 4a, the curves exhibited the isotherm of type IV with a series of typical adsorption behaviours including monolayer adsorption, multilayer adsorption and capillary condensation, indicating the characteristics of mesopores within the as-prepared membranes^35, 36^. And the hysteresis between adsorption and desorption isotherms reveal that the relevant mesopores are open, thus it cause the difference of capillary force for N2 adsorption and desorption. The narrow H_3_ hysteresis loop during the high pressure regimes revealed that the mesopores are coniform and open, within no significant interruption between the capillary evaporation and condensation for N_2_^36^. The relevant Brunauer-Emmett-Teller (BET) surface area of initial GA-MNPs and L-MNPs were 42.14 and 37.52 m^2^ g^−1^, respectively. The slight decrease of surface area after immobilization was due to the increase of particle size.

The fascinating adsorption isotherms of as-prepared L-MNPs enabled us to deeply investigate their hierarchical rough structure. The immobilization induced structural reorganization was investigated by quantitatively fractal analysis. Fractal dimension (*D*) with the values of 2 and 3 described a perfectly flat and a totally irregular rough surface, respectively, and hence, the fractal information determined for MNP samples indicated the hierarchical surface roughness. The calculation of *D* was through the modified Frenkel-Halsey-Hill (FHH) theory of multilayer gas adsorption:^36, 37^

1 $$\mathrm{ln}(V/{V}_{{\rm{mono}}})=A[{\rm{lnln}}({p}_{0}/p)]+{\rm{constant}}$$

where, *V* is the amount of N_2_ adsorbed at each equilibrium pressure, *V*_mono_ is the amount adsorbed of monolayer coverage (calculated through the BET equation), and *p*_0_ is the saturation pressure. Thus, a plot of ln(*V*/*V*_mono_) versus ln(ln(*p*_0_/*p*)) displayed a linear character, and the slope A could be used to calculate *D* utilizing the expression: *D* = *A* + 3, which was according to the dominant forces of liquid-gas surface tension at high coverage^38, 39^. Fig. 4b presented the relevant FHH plots reconstructed from N_2_ adsorption isotherms, indicating two distinct linear regions according to monolayer and high coverage regions. Because the interface of mesopore adsorption and capillary condensation is controlled by the liquid-gas surface tension forces, the slopes over the high coverage regions were taken to ensure a reliable evaluation of *D* ^39, 40^. The resultant *D* values of GA-MNPs and L-MNPs were 2.796 and 2.672, respectively, revealing the typical surface fractal feature with irregular rough structure. It is noteworthy that the L-MNPs retained over 95% of the original *D* values after immobilization, indicating a highly rough surface which is of great importance for effective catalysis.

In order to prove the nanoparticles are Fe_3_O_4_ with comparatively high purity, the XRD pattern of GA-MNPs and L-MNPs were measured and compared, respectively. Figure 4c presented the diffraction peaks of L-MNPs from (220), (311), (400), (422), (511) and (440) are respectively observed at 2*θ = *30.123^◦^, 35.499^◦^, 43.122^◦^, 53.483^◦^, 57.040^◦^, 62.621^◦^, while the corresponding peaks of GA-MNPs appear at 2*θ = *30.081^◦^, 35.442^◦^, 43.119^◦^, 53.418^◦^, 57.000^◦^, and 62.581^◦^. These results corresponded to the standard diffraction spectra (JCPDS: 65–3107), which suggested that MNPs is Fe_3_O_4_ with comparatively high purity, and the conjugation process of L-MNPs derived from GA-MNPs in TX-100 reverse micelles does not result in the phase change of Fe_3_O_4_ nanoparticles^41^.

The FT-IR spectra of L-MNPs obtained in TX-100 reverse micelles were measured at various stages of immobilization. For the three particles, the strong absorption band around 580 cm^−1^ was ascribed to the Fe-O bond of Fe_3_O_4_, while the band around 3445 cm^−1^ was assigned to the stretching and bending vibrations of hydroxyl groups. It was seen from NH_2_-MNPs curve that the new peaks at 1111 cm^−1^ and 1026 cm^−1^ referring to stretching vibration of Si–O, which might give evidence of the existence of APTES (Figure S1). In addition, it was showed from Fig. 4d that the strong absorption peak of L-MNPs in the 1700–1600 cm^−1^ region, 1580–1510 cm^−1^ region, 1400–1200 cm^−1^ region and 1200–900 cm^−1^ region corresponded to the C = O stretching vibrations, N–H bending, C–N stretching vibrations and protein-associated sugar chains, respectively^42^. The characteristic adsorption bands were similar to that of *Candida rugosa* lipase, which confirmed the conjugation of lipase on GA-MNPs in TX-100 reverse micelles.

### Catalytic Performance for Esterification

**Figure 5:**
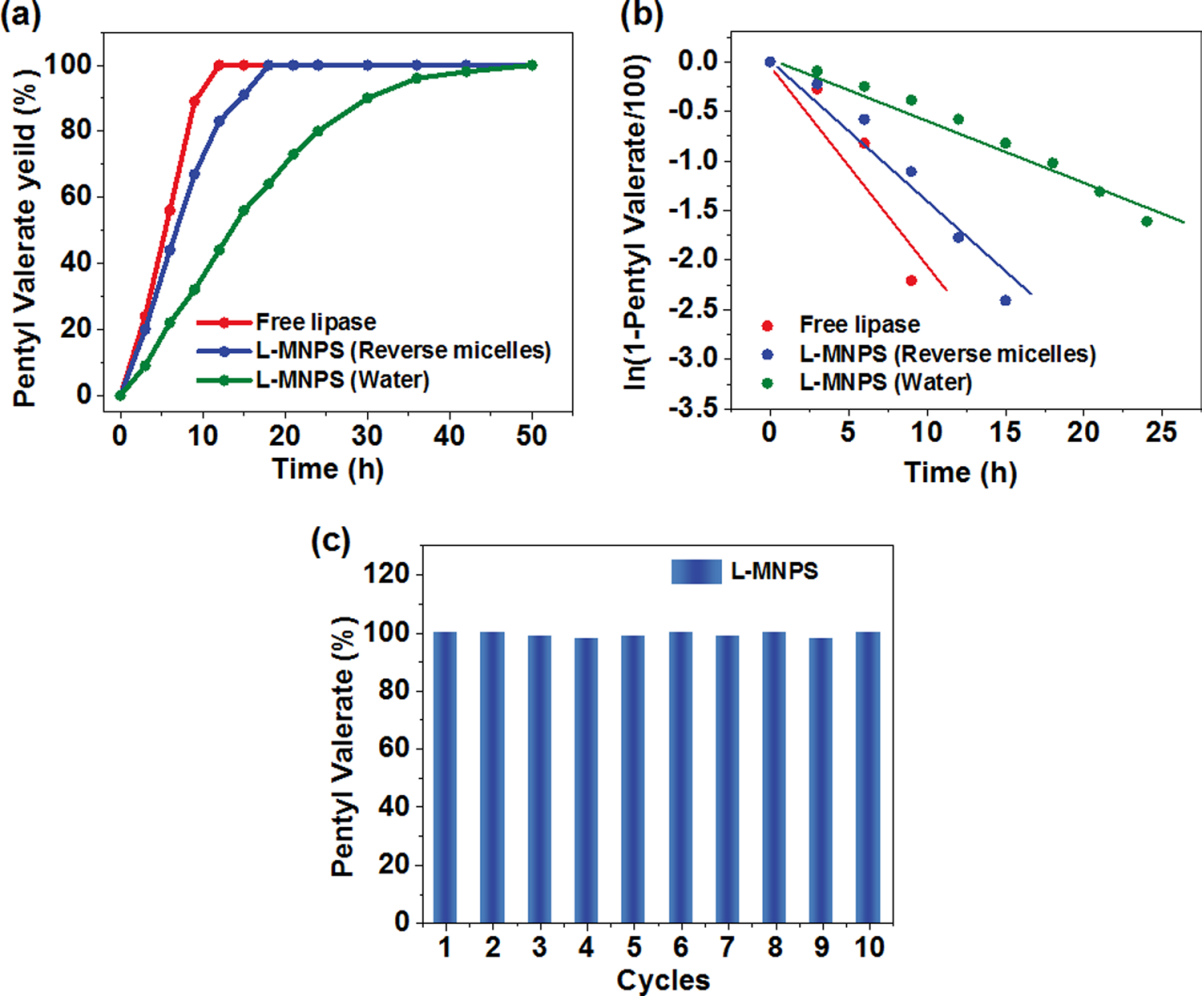
(**a**) Synthesis of pentyl valerate in cyclohexane using L-MNPs (synthesis from reverse micelles), L-MNPs (synthesis from water), and free *Candida rugosa* lipase. (**b**) The pseudo-first-order kinetic plot for synthesis of pentyl valerate for relevant samples. (**c**) Reversibility of the catalytic capacity for L-MNPs over 10 cycles.

The uniform and small sizes of the magnetic nanoparticles provided them with a high surface area-to-volume ratio, large density of binding sites for immobilization of enzyme, excellent dispersibility in water, fast motion with agitation, and rapid response toward magnetic field, which is of interest to their catalytic performance. As shown in Fig. 5a, in contrast to the poor activity of L-MNPs synthesis from common water system, the as-prepared L-MNPs from reverse micelle systems exhibit similar enzymatic activity with free *Candida rugosa* lipase, only slight decrease in catalytic efficiency was observed, highlighting the robust immobilization structure. Further quantitative relationship between the synthetic rate and the reaction time (*t*) for different *Candida rugosa* lipase was analyzed. A pseudo-first-order kinetic model was employed to fit the experimental data. The rate constant is defined as the slope of the plot for the equation for the first-order reaction^43, 44^.

2 $$\mathrm{ln}(1-{C}_{t}/{C}_{e})=-{k}_{1}t$$

where *k*_1_ is the rate constant and *t* is the reaction time (min), *C*_*t*_ and *C*_*e*_ are the synthetic rate of pentyl valerate at a time (*t*) and at equilibrium, respectively.

Figure 5b and Table 1 indicated that the synthetic process of pentyl valerate could be described by using a pseudo-first-order kinetic equation. The *k* obtained for L-MNPs synthesis from reverse micelles retained over 70% of the original enzymatic activity comparing with free *Candida rugosa* lipase, which was also increased over 150% than that of L-MNPs synthesis from common water system, demonstration the promising immobilization efficiency. This result was contributed by effective binding of enzyme molecules via hydrophobic interactions involving the active site which lead to a nonactive conformational change in enzyme structure or by spreading of enzyme on the Fe_3_O_4_ particles surface leading to an inactive configuration. Although immobilization of enzymes generally enhance their stability in bulk water, one major disadvantage of random immobilization of enzymes onto nanoparticles is that the activity of the immobilized enzyme is often significantly decreased because the active site may be blocked from substrate accessibility, multiple point-binding may occur, or the enzyme may be denatured. The nonionic reverse micelles were prepared and used as reaction medium for immobilization of *Candida rugosa* lipase, which avoid the random cross-linking between proteins and supports. Thus, the catalytic properties were improved. In order to evaluate the economic plausibility, it is very important for us to study the reusability of L-MNPs. We performed the reusability of L-MNPs for 10 times, and the influence of repetitive usage on catalytic capacity for synthesis of pentyl valerate was investigated. It was seen from Fig. 5c that the relatively stable catalytic capacity of L-MNPs was nearly constant even after being reused for 10 cycles, indicating the robust durability for long-term using.

**Table 1:** Results from linear regression of the plots for the pseudo-first-order kinetic equation.

Samples	*k*_1_	*R*
Free lipase	0.2389	0.9421
L-MNPs (from reverse micelles)	0.1695	0.9844
L-MNPs (from water)	0.0670	0.9764

### Magnetic Separation Performance

**Figure 6:**
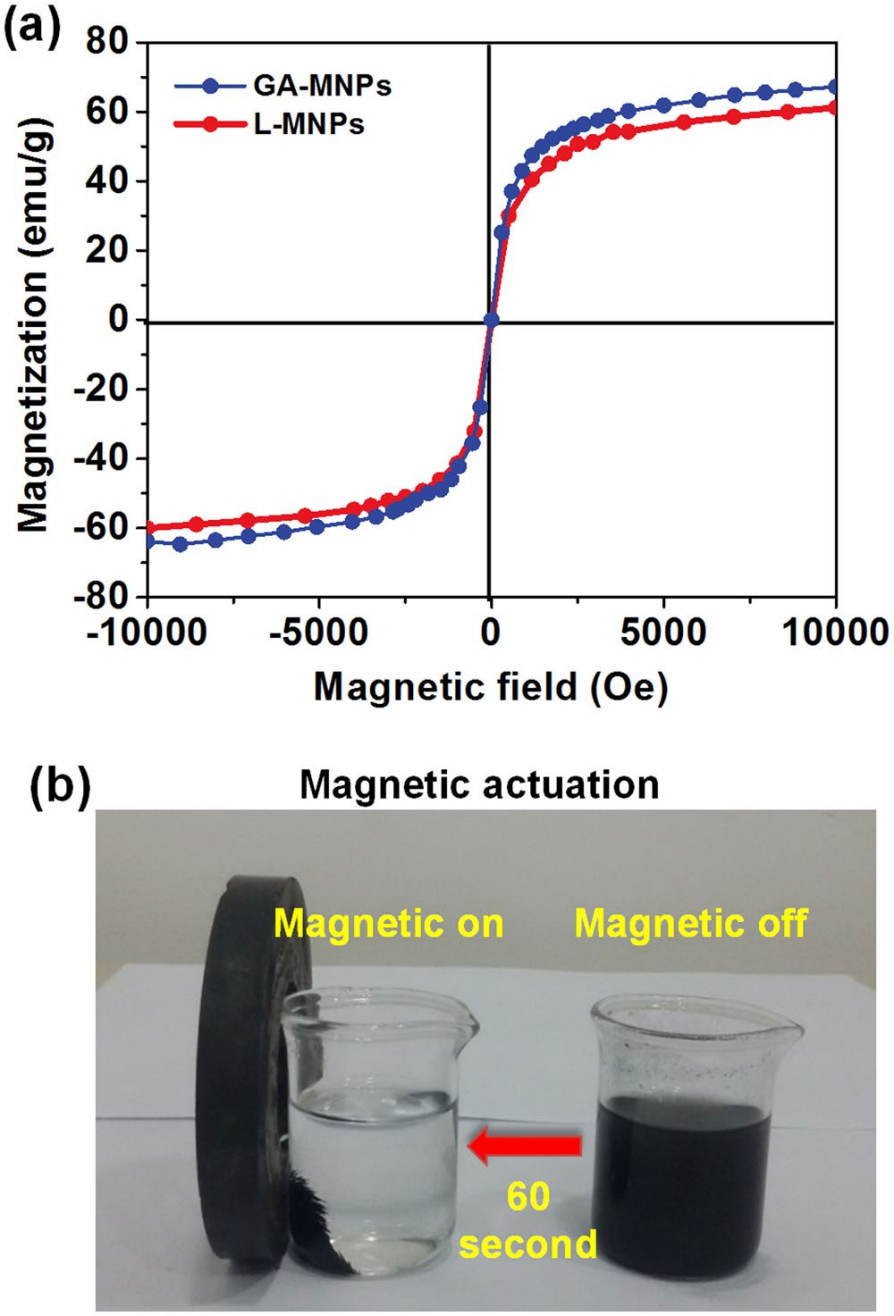
(**a**) The hysteresis loops of L-MNPs before and after immobilization. GA-MNPs. (**b**) Photograph showing the fast magnetic responsive performance of L-MNPs after catalytic reaction.

Figure 6a displayed the hysteresis loops of GA-MNPs and L-MNPs, which indicates they possess the magnetic saturation (*MS*) values of 65 emu g^−1^ and 59 emu g^−1^, respectively. It was worth noting that the remanence of these nanoparticles is zero once the applied magnetic field is removed, implying that these nanoparticles are superparamagnetic^45^. The superparamagnetic properties of the magnetic nanoparticles is critical for their application in biomedical and bioengineering field, which prevents them from aggregation and enables them to redisperse rapidly when the magnetic field is removed. These magnetically active properties of the L-MNPs render them very susceptible to magnetic fields and therefore make the solid L-MNPs and liquid phases separate easily without tedious separation processes (Fig. 6b), which is of great importance for real applications.

## Conclusions

In summary, we have presented scalable, synergistic assembly strategy for fabricating magnetic, hierarchical-structured L-MNPs by the combination of nonionic reverse micelle method and Fe_3_O_4_ nanoparticles. For the first time, the naturally abundant *Candida rugose* lipase was ordered-assemble into nanoparticles with high catalytic activity and durability. Benefiting from the high porosity, large surface area, fractal dimension, robust enzymatic activity, good durability, and high magnetic saturation (59 emu g^−1^), the L-MNPs effectively catalyze pentyl valerate esterification in a fast speed and facilely separated by an external magnet in 60 second. We expect that such fascinating L-MNPs will open up numerous opportunities for a wide range of applications in future industrial applications such as synthesis of biocatalysis, biomedical diagnostics, biosensors, microfluidics cells, and wastewater treatment.

## Experimental Section

### Materials and Reagents

Polyoxyethylene tert-octylphenyl ether (TX-100), n-octanol, isooctane, glutaric dialdehyde, 3-Aminopropyl triethoxysilane (APTES), 1-pentanol, and valeric acid were supplied by Chongqing Taixin Chemical Reagent Co. Ltd, China. Fe_3_O_4_ magnetic nanoparticles (MNPs, 15~30 nm) were supplied from Aladdin Chemical Co. Ltd, China. *Candida rugosa* lipase (type VII) was purchased from Sigma-Aldrich, St. Louis, MO, USA. Pure water with a resistance of 18.2 MΩ was obtained from a Millipore system.

### Preparation of Reverse Micelles

TX-100 and n-octanol with a 1:6 molar ratio were dissolved in isooctane at room temperature (TX−100 concentration of 0.2 mol L^−1^), and then a given volume of aqueous solution was dropped into the mixture using a microsyringe. After the injection, the mixture was vigorously stirred and treated using an ultrasonic mixer until it became a transparent reverse micelle system. The quantity of solubilized aqueous solution in the reverse micelle was shown by the molar ratio (*W*) of injected aqueous solution to TX−100, that is, *W* = [H_2_O]/[TX−100]^46, 47^. The structure of TX−100 reverse micelle was shown in Fig. 1.

### Fabrication of Glutaric Dialdehyde Modified MNPs

Figure 1a Describes the synthesis pathway. Generally, the MNPs were put in a mixture of 75 mL of water and 50 mL of ethanol. In order to disperse completely, the mixed solution was ultrasonically agitated for 0.5 h. After that, a certain amount of APTES was added. Then the mixture solution was kept stirring for 40 h before separation. The MNPs modified with APTES (NH_2_-MNPs) were prepared and washed with ethanol for 5 times. Subsequently, the NH_2_-MNPs was added in the 20 mL ethanol, then mixed with 25 mL glutaric dialdehyde (25%) and kept at 200 °C for 10 h with continuous agitation (200 rps). Finally, the NH_2_-MNPs activated with glutaric dialdehyde (GA-MNPs) were and obtained and washed with ethanol and water for 4 times, respectively.

### Immobilization of Lipase on GA-MNPs

As shown in Fig. 1b, the immobilization of lipase on GA-MNPs was carried out in TX-100 reverse micelles. 50 μL of *Candida rugose* lipase solution (3 mg mL^−1^) was mixed with 50 μL of GA-MNPs solution. The mixed aqueous solution was added in the 50 mL of TX-100 reverse micelles (*W = *10). Thereafter, the mixture was vigorously stirred and treated using an ultrasonic mixer until it became a transparent reverse micelle system. Finally, the L-MNPs were obtained and separated by an external magnetic field. For compassion with traditional synthesis method using water reaction medium, the 50 μL of lipase solution (3 mg mL^−1^) and 50 μL of GA-MNPs solution were added in 5 mL of Tris-HCl buffer (pH = 8.5). The mixture was shaken at 30 °C for 2 h. Finally, the L-MNPs were obtained and separated by an external magnetic field.

### Measurement of the Catalytic Performance

The catalytic performance of L-MNPs obtained in reverse micelles and in bulk water were tested for synthesis of pentyl valerate. After immobilization, the reaction system consisted of 2 mL of cyclohexane supplemented with equimolar (100 mM) concentrations each of 1-pentanol and valeric acid in 10 mL screw capped bottles. The reaction was initiated by addition of L-MNPs and shaking at 37 °C and 200 rpm in an orbital shaker. Esterification reaction using free enzyme equivalent to the amount immobilized on MNPs was also performed. The samples were withdrawn at regular intervals and the quantification of accumulated ester was analyzed on Agilent Technologies 6890 N network GC systems, USA, with a flame ionization detector^21^.

### Characterization

^1^H NMR spectra of TX-100 reverse micelles with different *W* values was conducted on an Inava 500MHZ NMR spectrometer (Varian Corporation, USA). FT-IR spectra of the water existed in reverse micelles with different *W* values was recorded over the range of 1000–4000 cm^−1^ with a Nicolet 8700 FT-IR spectrometer. The particle size distribution of the L-MNPs was determined with Particle Size Analyzer (DelsaTM Nano C Beckman Coulter, USA). The Brunauer–Emmet–Teller (BET) surface area was characterized using N_2_ adsorption–desorption isotherms with a surface area analyzer (ASAP 2020, micromeritics Co., USA). The morphology and structure of the L-MNPs were observed using transmission electron micrograph (TEM) with 120 kV (JEM-1200EX). The crystalline state of the L-MNPs was measured by X-ray diffractometer (Ultima IV; Rigaku, Tokyo, Japan). The imaging of L-MNPs for AFM was performed using a Nanoscope Multimode Scanning Probe Microscope equipped with an EV scanner operating in tapping mode. The magnetic measurements were carried out with a superconducting quantum interference device model MPMS Xl-7 magnetometer (USA) at a maximum magnetic field of 7 T and a sensibility of 10^–6^ emu.

## Electronic supplementary material

Supplementary Information
