# Supplementary material for: A reverse micelle strategy for fabricating magnetic lipase-immobilized nanoparticles with robust enzymatic activity
Source: Sci Rep. 2017 Aug 29;7:9806. doi: 10.1038/s41598-017-10453-4 (PMC5575323; doi:10.1038/s41598-017-10453-4)
Supplement: Supplementary file 1 — Supplementary Information [file 41598_2017_10453_MOESM1_ESM.doc]

Supporting Information

**A reverse micelle strategy for fabricating magnetic lipase-immobilized nanoparticles with robust enzymatic activity**

Shixiong Yi,^1^ Fangyin Dai,^1^ Cunyi Zhao,^2^ and Yang Si^2^,*

^1^State Key Laboratory of Silkworm Genome Biology & College of Biotechnology, Southwest University, Chongqing, 400715, P.R. China, and ^2^Fiber and Polymer Science, University of California, Davis, CA, 95616, USA.


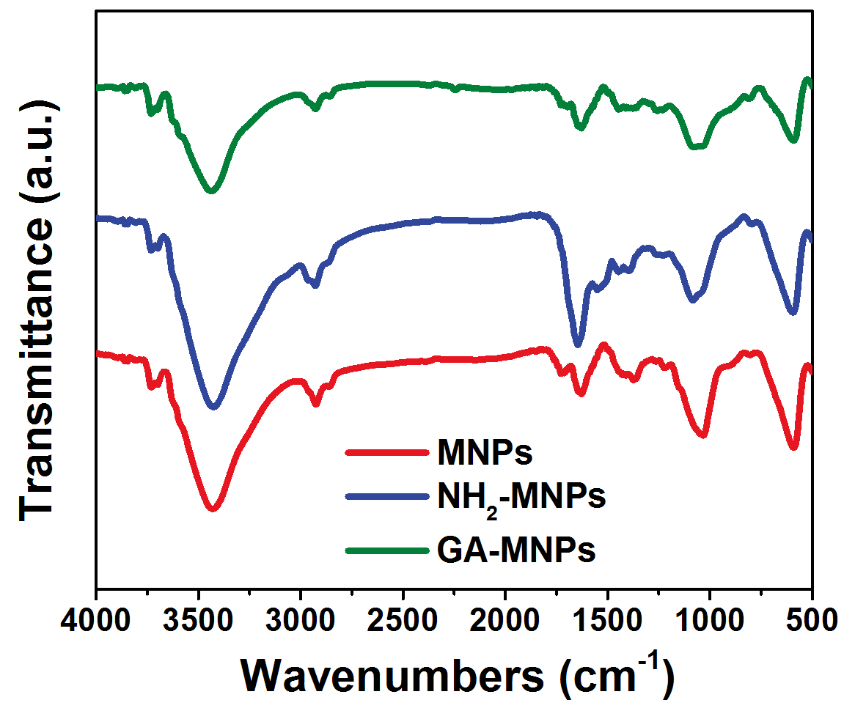


**Figure S1.** FT-IR spectra of MNPs, NH_2_-MNPs, and GA-MNPs
